# Population pharmacokinetics of total and unbound concentrations of intravenous posaconazole in adult critically ill patients

**DOI:** 10.1186/s13054-019-2483-9

**Published:** 2019-06-06

**Authors:** Fekade B. Sime, Catherine J. Byrne, Suzanne Parker, Janine Stuart, Jenie Butler, Therese Starr, Saurabh Pandey, Steven C. Wallis, Jeffrey Lipman, Jason A. Roberts

**Affiliations:** 1 School of Pharmacy, Centre for Translational Anti-infective Pharmacodynamics, The University of Queensland, Brisbane, Australia; 2 School of Pharmacy and Pharmaceutical Sciences, Trinity College Dublin, Dublin, Ireland; 3 University of Queensland Centre for Clinical Research, Faculty of Medicine, The University of Queensland, Building 71/918, Herston Rd, Herston, Queensland 4029 Australia; 4 Department of Intensive Care Medicine, Royal Brisbane and Women’s Hospital, Brisbane, Australia; 5 Pharmacy Department, Royal Brisbane and Women’s Hospital, Brisbane, Australia

**Keywords:** Intravenous posaconazole, Unbound pharmacokinetics, Critically ill, Antifungal

## Abstract

**Background:**

The population pharmacokinetics of total and unbound posaconazole following intravenous administration has not yet been described for the critically ill patient population. The aim of this work was, therefore, to describe the total and unbound population pharmacokinetics of intravenous posaconazole in critically ill patients and identify optimal dosing regimens.

**Methods:**

This was a prospective observational population pharmacokinetic study in critically ill adult patients with presumed/confirmed invasive fungal infection. A single dose of 300 mg posaconazole was administered intravenously as an add-on to standard antifungal therapy, and serial plasma samples were collected over 48 h. Total and unbound posaconazole concentrations, measured by chromatographic method, were used to develop a population pharmacokinetic model and perform dosing simulations in R using Pmetrics.

**Results:**

From eight patients, 93 pairs of total and unbound concentrations were measured. A two-compartment linear model with capacity-limited plasma protein binding best described the concentration-time data. Albumin and body mass index (BMI) were included as covariates in the final model. Mean (SD) parameter estimates for the volume of the central compartment (*V*) and the elimination rate constant were 72 (43) L and 42.1 (23.7) h^−1^, respectively. Dosing simulations showed that high BMI was associated with a reduced probability of achieving target total and unbound posaconazole concentrations. Low serum albumin concentration was associated with a reduced probability of attaining target total but not unbound posaconazole concentrations.

**Conclusions:**

An important clinical message of this study is that critically ill patients with increased BMI may require larger than approved loading doses of intravenous posaconazole when considering currently recommended dosing targets. Variability in plasma albumin concentration appears unlikely to affect dosing requirements when the assessment is based on unbound concentrations. Where available, therapeutic drug monitoring of unbound concentrations may be useful.

## Background

Posaconazole is a triazole antifungal agent with an extended-spectrum of activity against various yeasts and moulds [1]. It was initially marketed as an oral suspension that exhibited unpredictable pharmacokinetics related to variable bioavailability and later formulated into a sustained-release tablet to improve oral bioavailability [2]. However, the use of these oral preparations is likely to pose problems in patients with gut dysfunction. To avoid these bioavailability problems, an intravenous formulation of posaconazole was recently developed [3].

Clinical and pharmacokinetic evaluations that have defined dosing regimens for this new intravenous formulation were conducted mainly in the healthy volunteers, haematology patients or transplant recipients [4, 5]. However, the product is approved for treatment-refractory cases of invasive fungal infections, which can occur in critically ill patients. The lack of pharmacokinetic data derived from critically ill patients means that uncertainty remains on the adequacy of the approved doses for this patient population.

We have recently studied the single-dose pharmacokinetics of total posaconazole concentration in critically ill patients, in which we observed different exposures than previously described in healthy volunteers [6]. However, it was not clear whether critical illness, or other common clinical factors, such as altered plasma protein binding, were responsible for the different pharmacokinetics observed in the study cohort. Based on biological plausibility, given posaconazole is a lipophilic drug, critical illness-related pathophysiologic factors are not expected to significantly alter its pharmacokinetics [7]. In addition, being predominately eliminated by faecal route [8], the usual clinical covariates related to either renal or hepatic function are unlikely to affect its pharmacokinetics. We thus hypothesised that altered distribution related to change in body fat composition could affect dosing requirements given the high lipid solubility of posaconazole. In addition, given the extensive binding of posaconazole (99%) to plasma proteins [9], we hypothesised that altered protein binding may occur in patients with altered albumin concentration, with variable effects on total and unbound posaconazole exposure. We, therefore, developed a chromatographic assay method for analysis of unbound posaconazole, to measure unbound concentrations and develop a population pharmacokinetic model describing both total and unbound posaconazole concentrations, as well as utilise such a model to predict doses associated with optimal exposure using in silico simulations. In this paper, we present the results of the population pharmacokinetic analysis and subsequent dosing simulations.

## Methods

### Study design and setting

This was a prospective observational population pharmacokinetic study of posaconazole administered intravenously to critically ill patients with presumed or confirmed fungal infections. The setting was a quaternary referral intensive care unit (ICU) at the Royal Brisbane and Women’s Hospital, Australia. The Hospital’s Human Research Ethics Committee (HREC/16/QRBW/377) and that of The University of Queensland (2016001354) granted ethical clearance.

### Patients

All patients admitted to the ICU at the study hospital during the study period were screened for eligibility. The inclusion criteria were age ≥ 18 years, admission for ICU care, the presence of suspected or confirmed fungal infection requiring systemic antifungal therapy, and the presence of central venous access for drug administration. The exclusion criteria were age < 18 years, pregnancy, prescription of drugs known to interact with posaconazole, use of posaconazole within the last 2 weeks prior to enrolment and any documented history of a drug reaction to triazole antifungals. Informed consent was obtained from participants or their next of kin.

### Posaconazole administration

A single dose of 300 mg intravenous posaconazole solution, diluted with 0.9% sodium chloride or 5% dextrose in water, was administered to each study participant by slow infusion over 90 min through a central venous catheter. The drug was infused through a 0.22 μm polyethersulfone (PES) or polyvinylidene difluoride (PVDF) filter. The single dose of posaconazole was administered for the study purpose, as an add-on to a full course of another antifungal drug prescribed for therapeutic purpose as part of the usual care and at the discretion of the attending physician.

### Sample collection

Fourteen blood samples (each 2 mL) were collected over 48 h from an arterial catheter. The sampling scheme was the first sample immediately before the commencement of the posaconazole infusion, then during infusion at 15 min, 45 min, 75 min and 90 min and subsequently at 3 h, 5 h, 8 h, 12 h, 18 h, 24 h, 30 h, 36 h and 48 h after the commencement of infusion. Lithium heparin tubes were used for sample collection. The plasma was separated by centrifugation (3000 rpm for 10 min) and frozen under − 80 °C for storage until assay of total and unbound concentrations.

### Clinical data collection

Clinical data were collected for each patient using an electronic case report form, which included patient demographics, diagnosis, clinical microbiology data (isolated organism, susceptibility and the minimum inhibitor concentration, MIC, when available), clinical chemistry (makers of renal and hepatic function and serum albumin level), illness severity scores (Acute Physiology and Chronic Health Evaluation II [APACHE II] score on ICU admission and the Sequential Organ Failure Assessment [SOFA] score), renal replacement therapy modality and settings (if any) and concomitant medications.

### Posaconazole assay

Total and unbound posaconazole concentrations were measured using a validated ultra-high performance liquid chromatography-tandem mass spectrometry (UHPLC-MS/MS) method. The total concentration assay methodology, using a Shimadzu 8030+ mass spectrometer (Kyoto, Japan) with a range of measurement of 0.02 to 5 mg/L (precision of 4.7, 2.7 and − 5.3% and accuracy of 8.7, 0.2 and − 2.5% at concentrations of 0.06, 0.4 and 4 mg/L) has been summarised elsewhere [6]. The unbound assay method was based on the chromatography and detection of the total assay, but used a Shimadzu 8050 mass spectrometer (Kyoto, Japan) to achieve a calibration range of 0.0005 to 0.1 mg/L (precision of 6.8, 3.3 and 5.4% and accuracy of − 3.9, 5.2 and 8.2% at concentrations of 0.0015, 0.01 and 0.08 mg/L spiked in ultracentrifugated plasma). Sample preparation involved ultracentrifugation of plasma using Centrifree devices (Merck Millipore, Tullagreen, Ireland) to separate the unbound fraction. The ultracentrifuged plasma (30 μL) was spiked with internal standard (posaconazole-[d4]) and mixed with methanol. An aliquot of 2 μL of the supernatant was injected onto the UHPLC-MS/MS. The assay method met the Food and Drug Administration (FDA) validation criteria for bioanalysis, with stability and storage conditions covering the sample conditions prior to receipt at the analytical laboratory, as well as during receipt and analysis at the analytical laboratory [10].

### Population pharmacokinetic modelling

The Pmetrics user interface in R for non-parametric adaptive grid (NPAG) algorithm was used to develop a population pharmacokinetic model. Both total and unbound posaconazole concentration-time data were included in the model building together with available covariates.

### Structural base model and binding model

Initially, one- and two-compartment, linear and capacity-limited protein-binding models were fitted to total and unbound posaconazole concentrations simultaneously. Elimination from the central compartment and intercompartmental distribution were modelled as first-order processes.

For the linear binding models, unbound posaconazole concentrations were related to total concentrations by Eq. 1.

1 $$ {C}_{\mathrm{free}}={C}_{\mathrm{total}}\times \mathrm{FF}\times \frac{{\mathrm{Alb}}_{\mathrm{median}}}{\mathrm{Alb}} $$

where *C*_free_ and *C*_total_ are the unbound and total posaconazole concentrations (mg/L) respectively, FF is the free fraction of posaconazole, Alb is the plasma albumin concentration (g/L) and Alb_median_ is the median Alb for the study population.

For the capacity-limited (Michaelis-Menten type) binding models, unbound posaconazole concentrations were related to total concentrations by Eqs. 2–5, assuming that albumin is the sole binding protein for posaconazole in the plasma and that all binding sites have the same affinity for posaconazole.

2 $$ {C}_{\mathrm{bound}}=\frac{B_{\mathrm{max}}\times {C}_{\mathrm{free}}}{K_D+{C}_{\mathrm{free}}} $$

3 $$ {B}_{\mathrm{max}}=\mathrm{Alb}\times N\times \frac{M_{\mathrm{posa}}}{M_{\mathrm{Alb}}}\times 1000 $$

4 $$ {K}_D=\frac{1}{K_A}=\frac{k_{\mathrm{off}}}{k_{\mathrm{on}}} $$

5 $$ {C}_{\mathrm{free}}={C}_{\mathrm{total}}-{C}_{\mathrm{bound}} $$

where *C*_total_, *C*_bound_ and *C*_free_ are the total, bound and free plasma posaconazole concentrations (mg/L), respectively, *B*_max_ is the maximum binding concentration of posaconazole (mg/L), *N* is the number of posaconazole binding sites per molecule of albumin, *M*_posa_ is the molecular weight of posaconazole, *M*_Alb_ is the molecular weight of albumin, *K*_*D*_ is the equilibrium dissociation constant (mg/L), *K*_*A*_ is the equilibrium affinity constant (L/mg), *k*_off_ is the first-order dissociation rate constant (h^−1^) and *k*_on_ is the second-order association rate constant (L/mg/h). *N* was assumed to be 1.

### Error model

Based on the standard deviation (SD) of observations ([obs]), either a multiplicative (Error = SD*γ) or an additive (Error = [SD^2^ + *λ*^2^]^0.5^) error model was tested with each of the structural base models. In addition, assay error was modelled as a linear function (Error = C0 + C1*[obs]) starting with a generic set of coefficients, followed by iterative optimization.

### Development of the covariate model

Available clinical covariates were tested on structural model parameters of volume of distribution (*V*) and total clearance (CL). Tested covariates included age, gender, height, weight, body mass index (BMI), albumin, serum creatinine, creatinine clearance (urinary), presence of renal replacement therapy, alanine aminotransferase, aspartate aminotransferase, alkaline phosphatase, bilirubin, gamma-glutamyltransferase and SOFA score. If the inclusion of the covariate resulted in a statistically significant improvement in the log-likelihood value (*p* < 0.05) and/or improved the goodness-of-fit plots, it was supported for inclusion in the final model.

### Model evaluation

Model evaluation was performed by visual inspection and statistical evaluation of goodness of fit via the combination of diagnostic plots and objective functions metrics. Scatter plots of observed-versus-predicted concentrations were examined together with model bias and imprecision metrics. Bias was defined as the mean weighted error of predicted minus observed concentrations, *Σ* (predicted-observed/standard deviation)/*N*, and imprecision was defined as the bias-adjusted, mean weighted squared error of predicted minus observed concentration, i.e. *Σ*[(predicted-observed)^2^/(standard deviation)^2^]/*N* - *Σ* (predicted-observed)/standard deviations/*N*, where *N* is the number of observations/predictions. Scatter and histogram plots of residuals versus predicted-concentration or time were also examined. Normality of residual distribution was evaluated with D’Agostino test. The objective functions examined were the log-likelihood ratio (LLR) test for the nested models, Akaike information criterion (AIC) and Bayesian information criterion (BIC). The LLR chi-squared test within Pmetrics was used for statistical comparison of nested models with *p* < 0.5 considered as significant.

### Dosing simulations

Monte-Carlo dosing simulations (*n* = 1000) were performed using the final covariate model over the same period as the original sample collection, 48 h. Given the previously recommended targets of total steady-state concentration ≥ 0.7 mg/L for prophylaxis and ≥ 1 mg/L for treatment [11], and studies suggesting that posaconazole trough concentrations on day 2 post dose-commencement are approximately half of steady-state concentrations [12, 13], trough concentrations of 0.35 mg/L and 0.5 mg/L at 48 h were considered as a surrogate targets for prophylaxis and treatment, respectively. These total trough concentration targets correspond to unbound trough concentration targets of 0.0023 mg/L (prophylaxis) and 0.0033 mg/L (treatment) at 48 h, respectively, based on the mean free fraction for posaconazole of 0.65% in study patients. These trough targets were chosen to determine the probability of target attainment (PTA) for various simulated posaconazole dosage regimens. PTA was also determined based on the area under the total concentration-time cure (AUC) and the area under the free-concentration-time curve (*f*AUC) from 24 to 48-h post dose, normalised to the MIC. Considering previously recommended total AUC/MIC ratios for posaconazole of 100 for prophylaxis and 200 for treatment of fungal infections [13], *f*AUC/MIC targets of 0.65 (prophylaxis) and 1.3 (treatment) were used based on the mean free fraction of 0.65%. Simulated dosage regimens included loading doses of 300 to 800 mg given either hourly or 12 hourly for 24 to 48 h.

## Results

### Patient demography and clinical data

Eight critically ill patients were enrolled in the study. Table 1 summarises patient demography and relevant clinical data.

**Table 1:** Characteristics of study participants

Characteristic	*n* (%) or median (IQR)
Age (years)	46 (40–51)
Sex	
Male	7 (88%)
Female	1 (12%)
Body mass index (kg/m^2^)	22.6 (20.2–29.7)
Weight (kg)	68 (65–82)
Serum creatinine (μmol/L)	106 (78–197)
Urinary creatinine clearance (mL/min)	74 (53–109)
Albumin (g/L)	20 (18–24)
Alanine transaminase (IU/mL)	53 (28–60)
Aspartate transaminase (IU/mL)	47 (38–130)
Alkaline phosphatase (IU/mL)	75 (63–108)
Total bilirubin (μmol/L)	11 (10–20)
APACHE II score (admission)	17 (17–24)
SOFA Score	
Day 1	5 (3–6)
Day 2	3 (2–4)
Patients with positive culture	4 (50%)
Organisms isolated	
*Candida albicans*	2 (25%)
*Candida dubliniensis*	2 (25%)
*Candida parasilosis*	1 (12%)
*Candida glabrata complex*	1 (12%)
*Candida* spp.	1 (12%)
Antifungals prescribed	
*Fluconazole*	6 (75%)
*Voriconazole*	2 (25%)
*Caspofungin*	3 (37%)
*Amphotericin*	1 (12%)

*IQR* interquartile range, *APACHE II* Acute Physiology and Chronic Health Evaluation II, *SOFA* Sequential Organ Failure Assessment

### Plasma protein binding

The median (interquartile range, IQR) unbound fraction estimated from 93 pairs of total and unbound concentrations was 0.55% (0.36–1.9%). The mean (±SD) unbound fraction was 0.65% (± 0.39%). Coefficient of variation for the unbound fraction was 58.5%.

### Pharmacokinetic model building

**Fig. 1:**
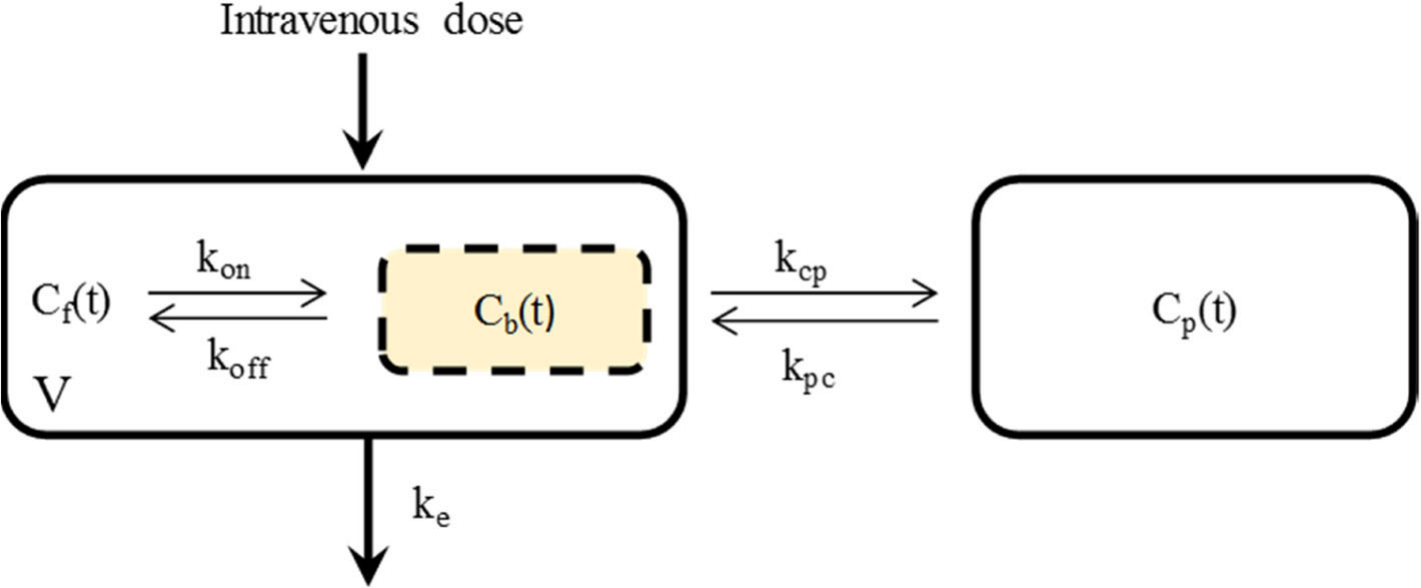
Schematics of the final structural pharmacokinetic model. *C*_*f*_(*t*) and *C*_*b*_(*t*) are free and bound posaconazole concentration in the central compartment at time *t*, respectively. *C*_*p*_(*t*), posaconazole concentration in the peripheral compartment at time *t*; *k*_*e*_, first-order elimination rate constant; *V*, volume of distribution of the central compartment; *k*_cp_, rate constant for distribution of unbound posaconazole from central to peripheral compartment; *K*_pc_, rate constant for distribution of unbound posaconazole from peripheral to central compartment; *K*_on_, second-order association rate constant for binding of posaconazole to albumin; *K*_off_, first-order rate constant for dissociation of posaconazole from albumin

**Fig. 2:**
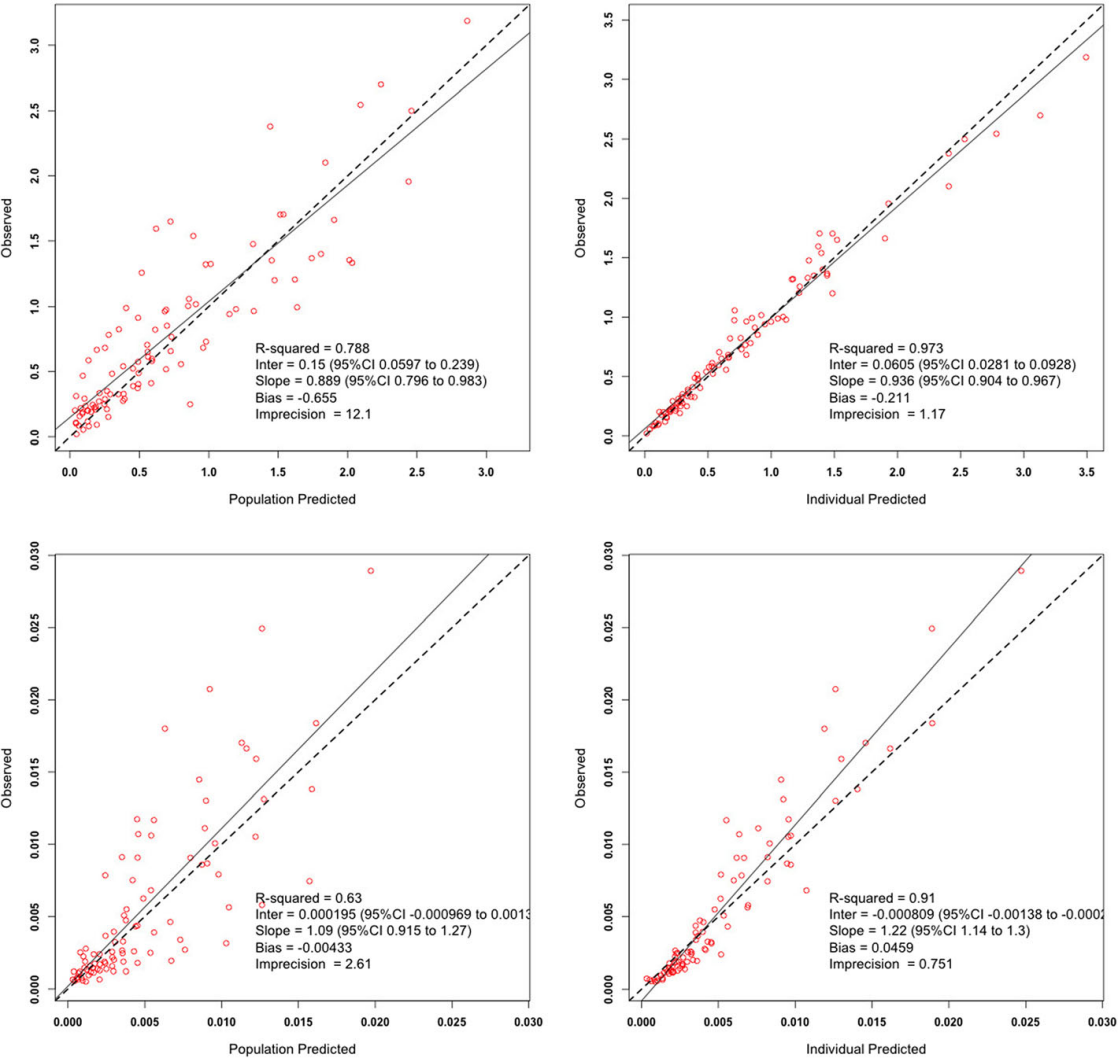
Observed-versus-predicted goodness-of-fit plots for total (top) and unbound (bottom) concentration

A two-compartment linear model with capacity-limited plasma protein binding best described the concentration-time data (Fig. 1). The only covariates that improved the goodness of fit and significantly reduced the objective function were BMI for volume of distribution (*V*) and albumin for *B*_max_. BMI was best related to *V* linearly and normalised to 24 (i.e. *V* = *V*_*Ɵ*_ × BMI/24, where *V*_*Ɵ*_ is typical value of *V* and 24 is the median BMI of study patients). The goodness-of-fit plots for the final covariate model are given in Fig. 2. Table 2 presents the parameter estimates for the final covariate model.

**Table 2:** Pharmacokinetic parameter estimates for the final covariate model

	Mean	SD	CV%
*K*_*e*_ (h^−1^)	42.07	23.68	56
*V*_*Ɵ*_ (l)	72.19	43.14	60
*K*_cp_ (h^−1^)	334.27	236.42	71
*K*_pc_ (h^−1^)	0.37	0.11	31
*K*_on_ (L/mg/h)	2820.35	671.99	24
*K*_off_ (h^−1^)	3897.40	596.46	15

*SD* standard deviation, *CV* coefficient of variation, *K*_*e*_ elimination rate constant, *V*_*Ɵ*_ typical volume of distribution of the central compartment, *K*_cp_ rate constant for distribution of unbound posaconazole from central to peripheral compartment, *K*_pc_ rate constant for distribution of unbound posaconazole from peripheral to central compartment, *K*_on_ second-order association rate constant for binding of posaconazole to albumin, *K*_off_ first-order rate constant for dissociation of posaconazole from albumin

### Dosing simulations

**Fig. 3:**
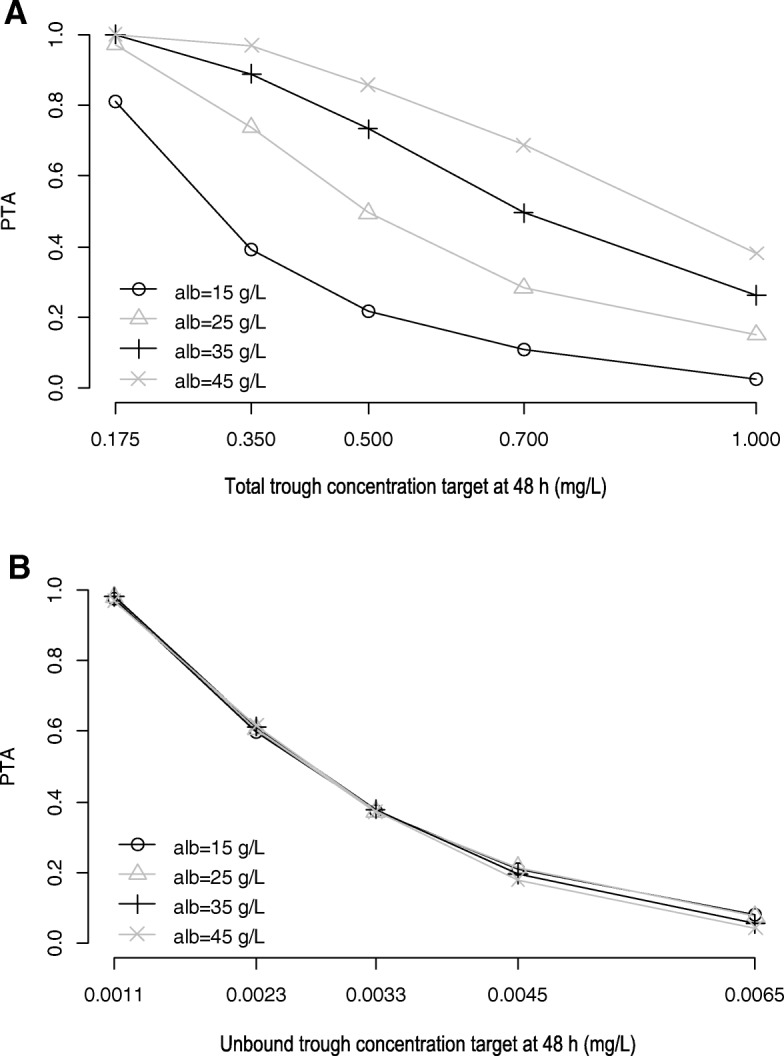
Probability of target attainment for a 300-mg intermittent intravenous infusion (90 min) of posaconazole given every 8 h for simulated patients with fixed BMI of 24 kg/m^2^ and varying albumin level considering total (**a**) and unbound (**b**) trough concentration targets

**Fig. 4:**
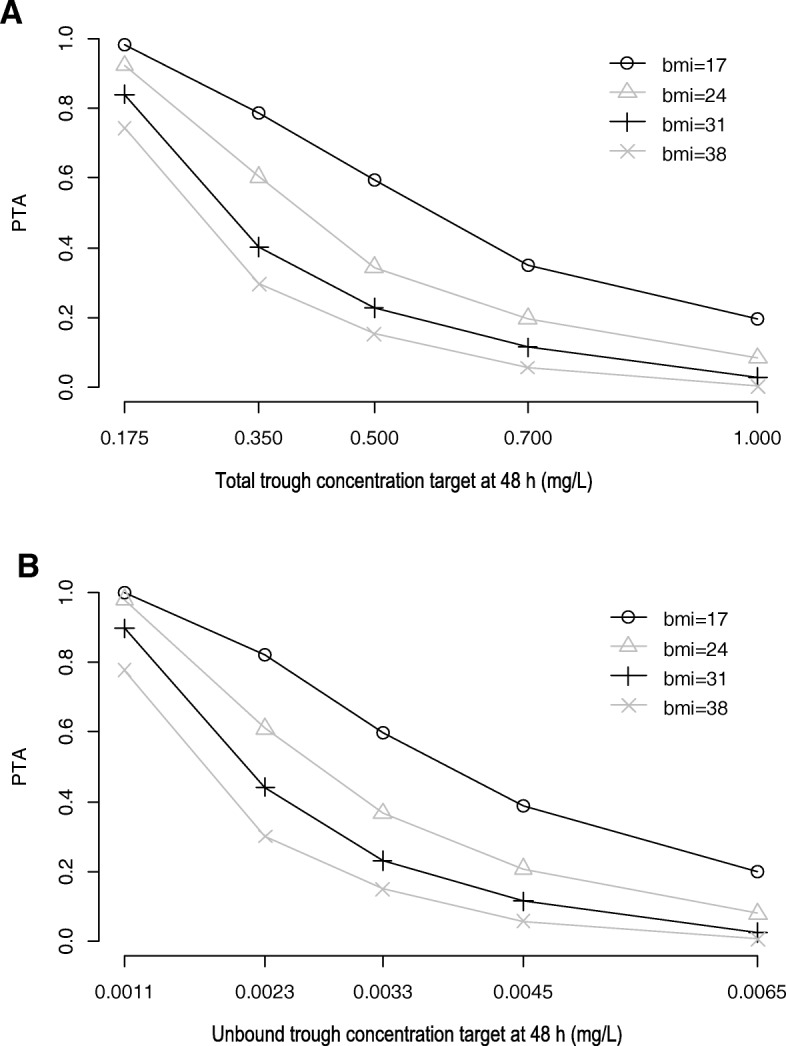
Probability of target attainment for a 300-mg intermittent intravenous infusion (90 min) of posaconazole given every 8 h for simulated patients with fixed albumin level of 20 g/L and varying BMI values considering total (**a**) and unbound (**b**) trough concentration targets

Loading dose regimens predicted to achieve target total and unbound trough concentrations at 48 h with a PTA of ≥ 80% are summarised in Tables 3 & 4 and 5 & 6. Generally, for both prophylaxis and treatment, an increase in BMI required an increased dose to achieve target total and unbound trough concentrations, whereas for a specific BMI value, a decrease in albumin concentration did not alter the dosing requirements when considering the unbound trough concentration target, but increased the dosing requirement when considering the total trough concentration target. This observation is further illustrated in Fig. 3a and b, whereby for a given dosing regimen of 300 mg IV every 8 h (as 90 min infusion) and fixed BMI value of 24 kg/m^2^, a decrease in albumin concentration does not change the PTA for unbound trough concentration target but decreases the PTA for the total trough concentration target. On the other hand, for the same dosing regimen and fixed albumin concentration of 20 g/L, Fig. 4 a and b illustrate that an increase in BMI is associated with reduced PTA for both total and unbound trough concentration targets. At the lowest albumin concentration of 15 g/L (Tables 3, 4, 5 and 6), lower doses were predicted for the unbound compared to the total trough concentration target for both treatment and prophylaxis across all BMI values tested.

**Table 3:** Intravenous loading dose regimens for prophylaxis, stratified by serum albumin concentration and body mass index, for ≥ 80% probability of achieving unbound trough concentration ≥ 0.0023 mg/L at 48 h

BMI (kg/m^2^)	Albumin (g/L)			
	15	25	35	45
17	300 mg q12h × 3	300 mg q12h × 3	300 mg q12h × 3	300 mg q12h × 3
24	500 mg q12 h × 3	500 mg q12h × 3	500 mg q12h × 3	500 mg q12h × 3
	400 mg q8h × 4	400 mg q8h × 4	400 mg q8h × 4	400 mg q8h × 4
31	600 mg q12h × 3	600 mg q12h × 3	600 mg q12h × 3	600 mg q12h × 3
	500 mg q8h × 4	500 mg q8h × 4	500 mg q8h × 4	500 mg q8h × 4
38	700 mg q12h × 3	700 mg q12h × 3	700 mg q12h × 3	700 mg q12h × 3
	600 mg q8h × 4	600 mg q8h × 4	600 mg q8h × 4	600 mg q8h × 4

**Table 4:** Intravenous loading dose regimens for prophylaxis, stratified by serum albumin concentration and body mass index, for ≥ 80% probability of achieving total trough concentration ≥ 0.35 mg/L at 48 h

BMI (kg/m^2^)	Albumin (g/L)			
	15	25	35	45
17	500 mg q12h × 3	300 mg q12h × 3	300 mg q12h × 3	300 mg q12h × 3
	400 mg q8h × 4			
24	600 mg q12h × 3	400 mg q12h × 3	300 mg q12h × 3	300 mg q12h × 3
	500 mg q8h × 4	300 mg q8h × 4		
31	800 mg q12h × 3	500 mg q12h × 3	400 mg q12h × 3	300 mg q12h × 3
	700 mg q8h × 4	400 mg q8h × 4	300 mg q8h × 4	
38	> 800 mg q12h × 3	600 mg q12h × 3	400 mg q12h × 3	300 mg q12h × 3
	800 mg q8h × 4	500 mg q8h × 4

**Table 5:** Intravenous loading dose regimens for treatment, stratified by serum albumin concentration and body mass index, for ≥ 80% probability of achieving unbound trough concentration ≥ 0.0033 mg/L at 48 h

BMI (kg/m^2^)	Albumin (g/L)			
	15	25	35	45
17	500 mg q12h × 3	500 mg q12h × 3	500 mg q12h × 3	500 mg q12h × 3
	400 mg q8h × 4	400 mg q8h × 4	400 mg q8h × 4	700 mg q8h × 4
24	600 mg q12h × 3	600 mg q12h × 3	600 mg q12h × 3	600 mg q12h × 3
	500 mg q8h × 4	500 mg q8h × 4	500 mg q8h × 4	500 mg q8h × 4
31	800 mg q12h × 3	800 mg q12h × 3	800 mg q12h × 3	800 mg q12h × 3
	700 mg q8h × 4	700 mg q8h × 4	700 mg q8h × 4	700 mg q8h × 4
38	> 800 mg q12h × 3	> 800 mg q12h × 3	> 800 mg q12h × 3	> 800 mg q12h × 3
	800 mg q8h × 4	800 mg q8h × 4	800 mg q8h × 4	800 mg q8h × 4

**Table 6:** Intravenous loading dose regimens for treatment, stratified by serum albumin concentration and body mass index, for ≥ 80% probability of achieving total trough concentration ≥ 0.5 mg/L at 48 h

BMI (kg/m^2^)	Albumin (g/L)			
	15	25	35	45
17	600 mg q12h × 3	400 mg q12h × 3	300 mg q12h × 3	300 mg q12h × 3
	500 mg q8h × 4	300 mg q8h × 4		
24	> 800 mg q12h × 3	500 mg q12h × 3	400 mg q12h × 3	300 mg q12h × 3
	700 mg q8h × 4	400 mg q8h × 4	300 mg q8h × 4	
31	> 800 mg q8h × 4	700 mg q12h × 3	500 mg q12h × 3	400 mg q12h × 3
		600 mg q8h × 4	400 mg q8h × 4	300 mg q8h × 4
38	> 800 mg q8h × 4	800 mg q12h × 3	600 mg q12h × 3	500 mg q12h × 3
		700 mg q8h × 4	500 mg q8h × 4	400 mg q8h × 4

Tables 7 and 8 summarise the ≥ 80% PTA when considering AUC/MIC ratio targets for either prophylaxis or treatment. Based on a desired PTA of ≥ 80%, a loading regimen of 300 mg by intermittent intravenous infusion (90 min) 12 hourly appears adequate for prophylactic cover when the presumed organism has a MIC value ≤ 0.12 mg/L. Higher MIC values of 0.25 mg/L and 0.5 mg/L would require at least 400 mg and 700 mg 8 hourly loading regimens, respectively (Tables 5 and 6). For treatment, 300 mg intravenous 8 hourly regimens appear adequate to cover organisms with MICs ≤ 0.063 mg/L, whereas for organisms with MICs ≥ 0.5 mg/L doses > 800 mg IV 8 hourly are needed to achieve the target AUC/MIC ratio with a probability of ≥ 80%.

**Table 7:** Monte-Carlo simulation predicted the probability of target attainment (PTA) for prophylaxis based on total and unbound AUC/MIC ratios of 100 and 0.65, respectively. Simulation was for albumin = 20 g/L and BMI = 24 kg/m^2^

Dosing regimen	PTA ≥ 80% for AUC/MIC of 100 by MIC (mg/L)					PTA ≥ 80% for *f*AUC/MIC of 0.65 by MIC (mg/L)				
	0.031	0.063	0.12	0.25	0.5	0.031	0.063	0.12	0.25	0.5
300 mg q8h × 4	✓	✓	✓	×	×	✓	✓	✓	×	×
400 mg q8h × 4	✓	✓	✓	✓	×	✓	✓	✓	✓	×
500 mg q8h × 4	✓	✓	✓	✓	×	✓	✓	✓	✓	×
600 mg q8h × 4	✓	✓	✓	✓	×	✓	✓	✓	✓	×
700 mg q8h × 4	✓	✓	✓	✓	×	✓	✓	✓	✓	✓
800 mg q8h × 4	✓	✓	✓	✓	✓	✓	✓	✓	✓	✓
300 mg q12h × 3	✓	✓	✓	×	×	✓	✓	✓	×	×
400 mg q12h × 3	✓	✓	✓	×	×	✓	✓	✓	×	×
500 mg q12h × 3	✓	✓	✓	×	×	✓	✓	✓	✓	×
600 mg q12h × 3	✓	✓	✓	✓	×	✓	✓	✓	✓	×
700 mg q12h × 3	✓	✓	✓	✓	×	✓	✓	✓	✓	×
800 mg q12h × 3	✓	✓	✓	✓	×	✓	✓	✓	✓	×

Check mark denotes PTA ≥ 80%; x mark denotes PTA < 80%

**Table 8:** Monte-Carlo simulation predicted the probability of target attainment (PTA) for treatment based on total and unbound AUC/MIC ratios of 200 and 1.3, respectively . Simulation was for albumin = 20 g/L and BMI = 24 kg/m^2^

Dosing regimen	PTA ≥ 80% for AUC/MIC of 200 by MIC (mg/L)					PTA ≥ 80% for *f*AUC/MIC of 1.3 by MIC (mg/L)				
	0.031	0.063	0.12	0.25	0.5	0.031	0.063	0.12	0.25	0.5
300 mg q8h × 4	✓	✓	×	×	×	✓	✓	×	×	×
400 mg q8h × 4	✓	✓	✓	×	×	✓	✓	✓	×	×
500 mg q8h × 4	✓	✓	✓	×	×	✓	✓	✓	×	×
600 mg q8h × 4	✓	✓	✓	×	×	✓	✓	✓	×	×
700 mg q8h × 4	✓	✓	✓	×	×	✓	✓	✓	✓	×
800 mg q8h × 4	✓	✓	✓	✓	×	✓	✓	✓	✓	×
300 mg q12h × 3	✓	✓	×	×	×	✓	✓	×	×	×
400 mg q12h × 3	✓	✓	×	×	×	✓	✓	×	×	×
500 mg q12h × 3	✓	✓	✓	×	×	✓	✓	✓	×	×
600 mg q12h × 3	✓	✓	✓	×	×	✓	✓	✓	×	×
700 mg q12h × 3	✓	✓	✓	×	×	✓	✓	✓	×	×
800 mg q12h × 3	✓	✓	✓	×	×	✓	✓	✓	×	×

Check mark denotes PTA ≥ 80%; x mark denotes PTA < 80

## Discussion

This is the first study describing the unbound population pharmacokinetics of intravenously administered posaconazole in critically ill patients. The results showed that BMI and serum albumin concentration appear to be important considerations for appropriate dosing of posaconazole.

When considering an unbound trough concentration target (Tables 3 and 5), an increase or decrease in albumin concentration does not alter the dosing requirements at a given BMI, because the free fraction would correspondingly decrease or increase, with the unbound concentration remaining unaffected. Thus, for a given adequate dosing regimen in a patient with a given BMI, variability in albumin concentration will not necessitate dose adaptation. This is clearly illustrated in Fig. 3 b where the PTA remains the same for a range of different albumin concentration for a dosing regimen. On the other hand, when considering the total trough concentration as a target, for a given BMI a decrease in albumin concentration predicted higher dosing requirements due to a reduction in PTA with decreased albumin concentrations (Fig. 3 a). This is consistent with the capacity-limited Michaelis-Menten type binding model that described the data set. Specifically, at trough concentrations which are much lower than the dissociation constant (*K*_*D*_), the unbound fraction (fu) can be estimated by fu = *K*_*D*_/(*B*_max_ + *K*_*D*_), and therefore, when albumin concentration decreases and thus *B*_max_ decreases (Eq. 3), the unbound fraction will be increased while the unbound concentration remains largely the same [14]. The total concentration will, however, be lower at lower albumin concentration consistent with the reduced extent of binding. Therefore, higher doses will be predicted by total trough concentration targets, consistent with the observation in Tables 3, 4, 5 and 6 for albumin level of 15 g/L. Of note, these doses are also higher than those predicted by the unbound trough concentration target. Put altogether, dosing prediction using total concentration targets would result in unnecessarily high doses in patients with hypoalbuminemia and progressively lower doses with increasing albumin concentration, which are sub-optimal, when compared to predictions with the unbound target. The clinical relevance of these results is that unbound but not total trough concentration targets should be used to determine dosing regimens of posaconazole or performing therapeutic drug monitoring guided dose adjustment when warranted [15]. Ideally, clinicians should rely on unbound concentration monitoring particularly in patients with marked hypoalbuminemia.

On the other hand, for a given albumin concentration, an increase in BMI predicted increased prophylactic or therapeutic dosing requirements with both total and unbound targets (Tables 3, 4, 5 and 6). Given posaconazole is a highly lipophilic drug, this observation is likely related to more extensive distribution into the adipose tissue with an increase in BMI resulting in increased *V* and thus decreased total concentration, which is also in agreement with a previous finding [16]. Since increased BMI (or obesity) is unlikely to affect the binding of posaconazole to albumin [17], the free fraction is expected to remain unaffected, and thus, a decrease in total concentration will subsequently result in a lower unbound concentration. Therefore, dose escalation appears necessary in obese patients even when using unbound trough concentration targets. Of note, using total concentration targets may give rise to more erroneous underprediction of dosing in patients with increased BMI and albumin concentration. For example, the underprediction was ≥ 50% in morbidly obese patients (BMI = 38 kg/m^2^) with normal albumin level (45 g/L) compared to about 17 to 20% in a patient with lower albumin level (25 g/L) and normal BMI (24 kg/m^2^) (Tables 3, 4, 5 and 6). Clinically, this would mean that patients with high BMI and normal albumin level are at a higher risk of underexposure even when receiving standard doses designed to achieve the conventional total concentration targets. Thus, unbound concentration monitoring may be particularly advantageous in obese patients even if their albumin concentration is well within the normal range.

Based on the AUC/MIC or *f*AUC/MIC treatment targets, loading regimens as high as 800 mg given 8 hourly for up to 4 doses (Table 8) were required to cover for wild type MIC distribution of *Aspergillus fumigatus* and *Aspergillus terreus* up to their epidemiologic cutoff (ECOFF) value of 0.25 mg/L. However, these doses were not adequate to cover the higher ECOFF (0.5 mg/L) of some *Aspergillus spp.* including *Aspergillus flavus*, *Aspergillus nidulans* and *Aspergillus niger* [11]. On the other hand for some of the most common *Candida spp*. with a relatively low ECOFF of 0.064 mg/L, including *Candida albicans*, *Candida dubliniensis*, *Candida parapsilosis* and *Candida tropicalis*, loading regimens as low as 300 mg 12 hourly for up to 3 doses appear adequate for ≥ 80% PTA. When comparing the 500 mg 8 hourly or 600 mg 12 hourly doses predicted by unbound trough concentration (Tables 5 and 6 for BMI 24), relative to the AUC/MIC based PTA in Table 7, only isolates with MIC ≤ 0.12 mg/L would be covered. Similarly, unbound trough concentration predicted prophylactic doses would only cover organisms with MIC ≤ 0.25 mg/L when assessed with AUC/MIC-based targets (Tables 3 and 4 vs Table 7 for BMI = 24 kg/m^2^). Thus, dosing predictions with trough concentration targets, without regard for MICs, may result in lower than required doses, particularly when high MIC organism is involved. However, since the susceptibility breakpoint of posaconazole has not yet been set [11] (although 0.5 mg/L had been proposed for aspergillosis [18]), it is difficult to conclusively comment on the adequacy of dosing based only on the ECOFF values. Furthermore, while the trough concentration targets were based on clinical analysis of outcomes [19–21], the AUC/MIC-based targets are yet to be validated in clinical studies. In addition, unbound serum concentrations far below the MIC were shown to have considerably better activity compared to the equivalent free concentration in a protein-free environment (as in the in vitro MIC test), which suggests that dosing in reference to MIC values may underestimate the clinical activity of posaconazole [22]. Thus, based on available evidence, doses predicted by the trough concentration targets inferred from clinical observations may be more clinically relevant.

We acknowledge that an important limitation of dosing predictions in this study is the lack of well-validated PK/PD dosing targets for posaconazole, particularly in relation to the MIC. Nonetheless, we considered steady-state trough concentration targets currently recommended by experts [11] although we inferred this from trough concentration at 48 h post dose commencement, as a surrogate for steady-state trough concentration. While we recognise the limitation of this, such surrogate measures of steady-state exposure at an earliest possible time point in the initial phase of therapy are also considered ideal for early concentration monitoring that aims for timely dose adjustment which would be a preferred approach in critically ill patients. Another important limitation is the small samples size, which offers a less diverse spread of covariates such as BMI for unequivocal extrapolation of the study findings.

## Conclusions

The critically ill patient population may require larger than currently approved loading doses of intravenous posaconazole to ensure an early and adequate steady-state exposure. Such dosing regimens should ideally be determined based on unbound concentration measurement because total trough concentration targets can give rise to erroneous dose prediction due to the extensive plasma protein binding. However, variability in plasma albumin concentration appears unlikely to affect dosing requirements when assessed based on unbound concentration. On the other hand, obesity may affect dosing requirements with relatively higher doses needed in those with high BMI. Thus, dosing in this population deserves further clinical investigation.

## Data Availability

The datasets for the current study are not publicly available due to ethics restrictions on the use of patient data but are available from the corresponding author given ethical clearance could be obtained to access these data.

## Abbreviations

APACHE II: Acute Physiology and Chronic Health Evaluation II
AUC: Area under the total concentration-time cure
BMI: Body mass index
CL: Clearance
*f*AUC: Area under the free-concentration-time curve
FDA: Food and Drug Administration
ICU: Intensive care unit
IQR: Interquartile range
LLR: Log-likelihood ratio
MIC: Minimum inhibitory concentration
NPAG: Non-parametric adaptive grid
PES: Polyethersulfone
PTA: Probability of target attainment
PVDF: Polyvinylidene difluoride
SD: Standard deviation
SOFA: Sequential Organ Failure Assessment
UHPLC-MS/MS: Ultra-high performance liquid chromatography-tandem mass spectrometry
*V*: Volume of distribution of the central compartment

## References

[1] Sabatelli F, Patel R, Mann PA (2006). In vitro activities of posaconazole, fluconazole, itraconazole, voriconazole, and amphotericin B against a large collection of clinically important molds and yeasts. Antimicrob Agents Chemother.

[2] Guarascio AJ, Slain D (2015). Review of the new delayed-release oral tablet and intravenous dosage forms of posaconazole. Pharmacother.

[3] Maertens J, Cornely OA, Ullmann AJ (2014). Phase 1B study of the pharmacokinetics and safety of posaconazole intravenous solution in patients at risk for invasive fungal disease. Antimicrob Agents Chemother.

[4] Cornely OA, Robertson MN, Haider S (2017). Pharmacokinetics and safety results from the phase 3 randomized, open-label, study of intravenous posaconazole in patients at risk of invasive fungal disease. J Antimicrob Chemother.

[5] Kersemaekers WM, van Iersel T, Nassander U (2015). Pharmacokinetics and safety study of posaconazole intravenous solution administered peripherally to healthy subjects. Antimicrob Agents Ch.

[6] Sime Fekade B., Stuart Janine, Butler Jenie, Starr Therese, Wallis Steven C., Pandey Saurabh, Lipman Jeffrey, Roberts Jason A. (2018). Pharmacokinetics of Intravenous Posaconazole in Critically Ill Patients. Antimicrobial Agents and Chemotherapy.

[7] Blot SI, Pea F, Lipman J (2014). The effect of pathophysiology on pharmacokinetics in the critically ill patient--concepts appraised by the example of antimicrobial agents. Adv Drug Deliv Rev.

[8] Krieter P, Flannery B, Musick T (2004). Disposition of posaconazole following single-dose oral administration in healthy subjects. Antimicrob Agents Ch.

[9] Andes D, Marchillo K, Conklin R (2004). Pharmacodynamics of a new triazole, posaconazole, in a murine model of disseminated candidiasis. Antimicrob Agents Chemother.

[10] Guidance for Industry, Bioanalytical Method Validation, U.S. Department of Health and Human Services, Food and Drug Administration, Center for Drug Evaluation and Research (CDER), Center for Veterinary Medicine (CMV), May 2018. Document available at https://www.fda.gov/downloads/drugs/guidances/ucm070107.pdf

[11] European Committee on Antimicrobial Susceptibility Testing. Posaconazole: rationale document for clinical breakpoints version 2.0 http://www.eucast.org., 2017.

[12] Green MR, Woolery JE (2012). Posaconazole serum level on day 2 predicts steady state posaconazole serum level. Ther Drug Monit.

[13] Dekkers BGJ, Bakker M, van der Elst KCM (2016). Therapeutic drug monitoring of posaconazole: an update. Curr Fungal Infect R.

[14] Toutain PL, Bousquet-Melou A (2002). Free drug fraction vs. free drug concentration: a matter of frequent confusion. J Vet Pharmacol Ther.

[15] Howard SJ, Felton TW, Gomez-Lopez A (2012). Posaconazole: the case for therapeutic drug monitoring. Ther Drug Monit.

[16] Miceli MH, Perissinotti AJ, Kauffman CA (2015). Serum posaconazole levels among haematological cancer patients taking extended release tablets is affected by body weight and diarrhoea: single centre retrospective analysis. Mycoses.

[17] Hanley MJ, Abernethy DR, Greenblatt DJ (2010). Effect of obesity on the pharmacokinetics of drugs in humans. Clin Pharmacokinet.

[18] Verweij PE, Howard SJ, Melchers WJ (2009). Azole-resistance in Aspergillus: proposed nomenclature and breakpoints. Drug Resist Updat.

[19] Jang SH, Colangelo PM, Gobburu JVS (2010). Exposure-response of posaconazole used for prophylaxis against invasive fungal infections: evaluating the need to adjust doses based on drug concentrations in plasma. Clin Pharmacol Ther.

[20] Felton TW, Baxter C, Moore CB (2010). Efficacy and safety of posaconazole for chronic pulmonary aspergillosis. Clin Infect Dis.

[21] Dolton MJ, Ray JE, Marriott D (2012). Posaconazole exposure-response relationship: evaluating the utility of therapeutic drug monitoring. Antimicrob Agents Chemother.

[22] Lignell A, Lowdin E, Cars O (2011). Posaconazole in human serum: a greater pharmacodynamic effect than predicted by the non-protein-bound serum concentration. Antimicrob Agents Chemother.

